# Accurate Inference of Tumor Purity and Absolute Copy Numbers From High-Throughput Sequencing Data

**DOI:** 10.3389/fgene.2020.00458

**Published:** 2020-04-30

**Authors:** Xiguo Yuan, Zhe Li, Haiyong Zhao, Jun Bai, Junying Zhang

**Affiliations:** ^1^School of Computer Science and Technology, Xidian University, Xi’an, China; ^2^School of Computer Science and Technology, Liaocheng University, Liaocheng, China; ^3^Department of Medical Oncology, Shaanxi Provincial People’s Hospital, Xi’an, China

**Keywords:** tumor purity, absolute copy numbers, high-throughput sequencing data, read depths, non-linear model

## Abstract

Inference of absolute copy numbers in tumor genomes is one of the key points in the study of tumor genesis. However, the mixture of tumor and normal cells poses a big challenge to this task. Accurate estimation of tumor purity (i.e., the fraction of tumor cells) is a necessary step to solve this problem. In this paper, we propose a new approach, AITAC, to accurately infer tumor purity and absolute copy numbers in a tumor sample by using high-throughput sequencing (HTS) data. In contrast to many existing algorithms for estimating tumor purity, which usually rely on pre-detected mutation genotypes (heterogeneity and homogeneity), AITAC just requires read depths (RDs) observed at the regions with copy number losses. AITAC creates a non-linear model to correlate tumor purity, observed and expected RDs. It adopts an exhaustive search strategy to scan tumor purity in a wide range, and chooses the tumor purity that minimizes the deviation between observed RDs and expected ones as the optimal solution. We apply the proposed approach to both simulation and real sequencing data sets and demonstrate its performance by comparing with two classical approaches. AITAC is freely available at https://github.com/BDanalysis/aitac and can be expected to become a useful approach for researchers to analyze copy numbers in cancer genome.

## Introduction

DNA copy number variation (CNV), as a type of structural variations, accounts for the majority of genomic mutations in cancers (McCarroll and Altshuler, 2007). Accurate detection of CNVs and quantification of absolute copy numbers provide insights into the progression of cancers and useful diagnostic indicators for cancer patients. High-throughput sequencing (HTS) technologies developed in recent years have generated a wealth of data for such tasks at base-pair resolution. A lot of computational approaches have been developed for CNV detection by using sequencing data (Yu et al., 2014; Zhang et al., 2015; Chen et al., 2017; Yuan et al., 2018b). However, contamination of normal cells in tumor tissues makes the observed magnitudes of signals in CNV regions diminished, which will lead to a decreased power in the detection of genomic mutations if the fraction is unknown (Yuan et al., 2012, 2017, 2018a). In addition, the contamination of normal cells in tumor tissues can also lead to adverse effects on subsequent genomic analysis, and further poses effects on patient’s condition analysis in clinical practice. Therefore, it is necessary and meaningful to develop computational methods for an accurate estimation of tumor purity and inference of absolute copy numbers.

Currently, there exist a number of methods to predict tumor purity by using either HTS data or SNP array data. For example, ABSOLUTE (Carter et al., 2012), as one of the most popular purity estimation methods, can work on both NGS and SNP array data sets. The central principle behind ABSOLUTE is that it incorporates segmented copy number data and prior probabilities from cancer karyotypes to search the best solution to tumor purity and tumor ploidy. BACOM (Yu et al., 2011) and AISAIC (Zhang et al., 2014) carried out tumor purity estimation based on SNP array data. The key idea behind them is that they utilize pre-detected copy number deletions (heterogeneous deletions or homogeneous deletions) to correlate tumor purity and observed copy numbers, and directly calculates tumor purity. Similarly, Sequenza (Favero et al., 2015) also relies on pre-detected heterozygous and homozygous deletions for tumor purity estimation. The difference is that Sequenza works on HTS data. In addition, PurityEst (Su et al., 2012) and PurBayes (Larson and Fridley, 2013) also perform tumor purity estimation by using HTS data. The common feature of them is that they require predetermined SNV heterozygous loci. Theoretically, mutation genotypes or karyotypes analyzed in previous step provide clear guidelines to create models for purity estimation. However, such types of information are not always available and the prediction of them is usually affected by many artifacts, such as sequencing errors and tumor impurity itself. Thus, it is very important to find a new method that does not rely on the pre-determined genotypes or karyotypes.

With the above considerations, in this article, we developed a new method, AITAC (Accurate Inference of Tumor purity and Absolute Copy number), toward inferring tumor purity and absolute copy numbers using HTS data. It utilizes the regions with copy number deletions, and models a non-linear relationship between the tumor purity and the observed read depths (RDs) and expected RDs in the regions. An exhaustive search strategy is adopted to find the optimal solution to the tumor purity from a wide range of values. Compared with existing methods for tumor purity estimation, AITAC has two distinguishing features: (1) it doesn’t require predetermined mutation genotypes, instead, it only requires genome regions with copy number deletions and utilizes them as the input for tumor purity prediction; (2) it models a non-linear relationship between tumor purity, observed RDs and expected RDs, and exhaustively searches the optimal solution to the tumor purity, which minimizes the deviation between observed RDs and expected RDs at the deleted regions.

To demonstrate the performance of the proposed method, we conduct simulation experiments to evaluate the accuracy of inferring tumor purity and absolute copy numbers, and make a comparison with existing methods. The results indicate that the AITAC method has its advantage. We further apply AITAC to a set of real sequencing samples from lung cancer patients and make a comparison with the estimates of ABSOLUTE (Carter et al., 2012), the results of which indicate that AITAC is a valid method. AITAC is written and implemented in Python. To make a complete analysis pipeline from sequencing data to a report on tumor purity and absolute copy numbers, we incorporate our previously developed CNV detection method, CNV_IFTV (Yuan et al., 2019b), into the AITAC algorithm. The source code of AITAC is available at https://github.com/BDanalysis/aitac and can be downloaded freely. We expect AITAC to be a useful tool for copy number analysis in biomedical research communities.

The reminder of this paper is organized as below. In Section “Materials and Methods”, we demonstrate the implementation of the AITAC method, including the major principles and technique. In Section “Results,” we conduct simulation studies to evaluate the performance of AITAC, and make a comparison to several peer methods. Furthermore, we apply AITAC to real sequencing data to validate and demonstrate its usefulness. Finally, we make a discussion and conclusion in Section “Discussion.”

## Materials and Methods

### Workflow and Rationale of the AITAC Method

**FIGURE 1:**
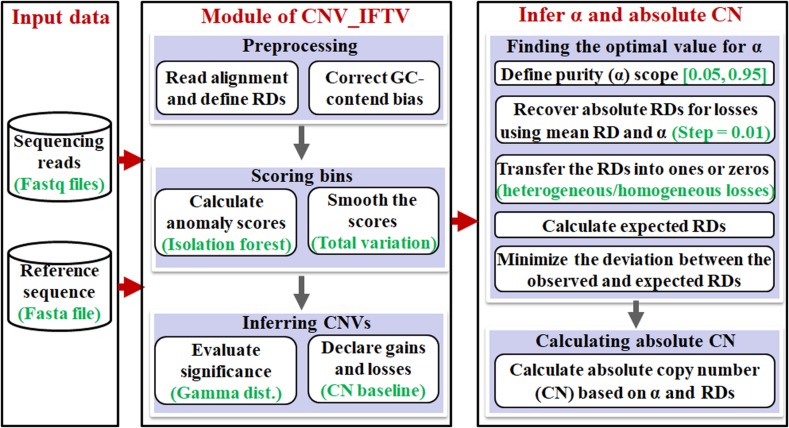
Workflow of the AITAC method for the inference of tumor purity and absolute copy numbers.

The workflow of the AITAC method is depicted in Figure 1. It starts with an input of a sequencing sample and a reference genome, and then performs a pipeline analysis via three primary steps: (1) detection of CNVs by using the CNV_IFTV (Yuan et al., 2019b) algorithm, (2) inference of tumor purity based on the regions with copy number detections, and (3) calculation of absolute copy numbers for all the CNV regions detected by CNV_IFTV. Here, the CNV_IFTV algorithm is based on isolation forest algorithm and total variation for the measurement of genome bins, and adopts a Gamma distribution for significance testing of the measurement (anomaly scores). One of the most important features of CNV_IFTV is that it captures the major characteristics of copy number data with the anomaly scores, which are non-linear mappings of RD signals. Flexibly, the module of the detection of CNVs using CNV_IFTV can be replaced by other approaches according to the users.

The rationale behind the AITAC method can be described briefly. The contamination of normal cells in tumor tissues can bring about a bias to the observation of copy numbers of tumor genomes (Yu et al., 2011). Accordingly, starting from the analysis of copy numbers can provide a clue for the estimation of tumor purity in tumor tissues. Since the copy number in human normal genome is supposed to 2, while the copy number in human tumor genome can be varied in a wide span (e.g., from 0 to 100), the combination of normal genome and tumor genome can be modeled theoretically, and can be further associated with the observations by creating a relationship model. Moreover, since deletions generally have two states: heterogeneity and homogeneity. Constructing models based deletion regions will be relatively simplified for the estimation of tumor purity. From this perspective, AITAC takes the detected deletion regions as the basis and creates a non-linear model to correlate tumor purity, observed and expected RDs, as so to obtain the purpose of estimating tumor purity. Based on this estimation, AITAC further infers absolute copy numbers.

In the following text, we first make a brief illustration to the CNV_IFTV algorithm, and then focus on the demonstration of the principles of the other two steps.

### Detection of CNVs

We choose our previously developed algorithm CNV_IFTV (Yuan et al., 2019b) for the CNV detection. The input data to the algorithm includes a human genome reference (e.g., HG19 or HG38) and a tumor genome with an aligned BAM file. The BAM file can be obtained from an alignment process by using the most popular algorithm BWA (Li and Durbin, 2009). Read count profile is extracted from the BAM file by using SAMtools (Li et al., 2009). Positions in the reference represented with “N”s are removed or filled with the average read count value. A RD profile is generated by dividing the whole genome into non-overlapping genome bins (e.g., bin size = 1000 or 2000 bp). As for the setting of the bin size, we choose such values, which are moderate in the detection of CNVs (Yuan et al., 2018a, 2019a). With the RD profile, GC-content bias across the whole genome is then corrected through a widely used approach introduced in the works (Abyzov et al., 2011). The key principle of the CNV_IFTV algorithm is summarized below.

With the preprocessed RD profile, CNV_IFTV detects CNVs via assigning an anomaly score to each genome bin and testing significance for the anomaly score (Yuan et al., 2019b). The anomaly score is created based on an isolation forest algorithm and a total variation model. Specifically, 256 isolation trees are established by randomly selecting 256 × 256 genome bins (256 genome bins for each isolation tree) from the genome being analyzed, and an anomaly score is assigned to each bin by comparing its RD to the isolation trees, and then the anomaly score is smoothed by total variation. More details are presented in Yuan et al. (2019b). The smoothed anomaly score is tested by using a Gamma distribution and the corresponding *p*-value is derived. Finally, a genome bin is declared as a CNV if the corresponding *p*-value is less than the pre-defined significance level (e.g., 0.05 or 0.01). Compared with the statistics of existing methods for CNV detection, the statistic used in AITAC has several distinct features: (1) it is a non-linear mapping of RDs, capturing two realistic characteristics of copy number data, i.e., a small fraction of regions in the whole genome are CNVs and the RDs of CNV regions in tumor genome are clearly different from those of normal regions; (2) it considers the intrinsic correlations among positions in the genome, which have a great impact on the analysis of copy number data since copy numbers are generally correlated in adjacent positions (Yuan et al., 2012). The performance of CNV_IFTV has been demonstrated in Yuan et al. (2019b) through a great number of experiments. The results indicate the advantages of CNV_IFTV. However, such module integrated in the AITAC method is allowed to be replaced by some more powerful algorithms for CNV detection.

### Inference of Tumor Purity

With the above detected CNVs, losses (*X*) of them are extracted to model a non-linear relationship among their observed RDs (*R*(*X*)), expected RDs (*E*[*R*(*X*)]), and the tumor purity (α). The reason of why only using copy number lost regions is that losses have only two states (i.e., heterogeneity and homogeneity), which can facilitate the establishment of the model. The non-linear relationship is expressed using the following Equations (Yu et al., 2011; Yuan et al., 2018a). Here, Equation (1) is a reflection of the relationship between the absolute RDs in tumor genome, RD in normal genome (the average RD across the whole cancer genome is approximately regarded as the RD value), and observed RDs in the mixture genomes.

(1) $$Q\,\left(X\right)=\frac{R\,\left(X\right)-\left(1-\alpha \right)\,\bar{r}}{\alpha }$$

(2) $$E\,\left[R\,\left(X\right)\right]=\frac{\alpha \,\bar{r}\,C\,\left(Q\,\left(X\right)\right)}{2}+\left(1-\alpha \right)\,\bar{r}$$

where $\bar{r}$ denotes the average RD across the cancer genome to be analyzed, *Q*(*X*) denotes the absolute RDs of the losses, and *C*(*Q*(*X*)) stands for a non-linear transformation of *Q*(*X*) to the value of either one (heterogeneous loss) or zero (homogeneous loss). Such transformation is carried out by clustering *Q*(*X*) into two groups. Subsequently, an exhaustive search strategy is used to find the optimal value for α, which minimizes the deviation between *R*(*X*) and *E*[*R*(*X*)]. Here, the range of α is set to be the interval [0.05, 0.95] and the step-size in search is set to 0.01.

**FIGURE 2:**
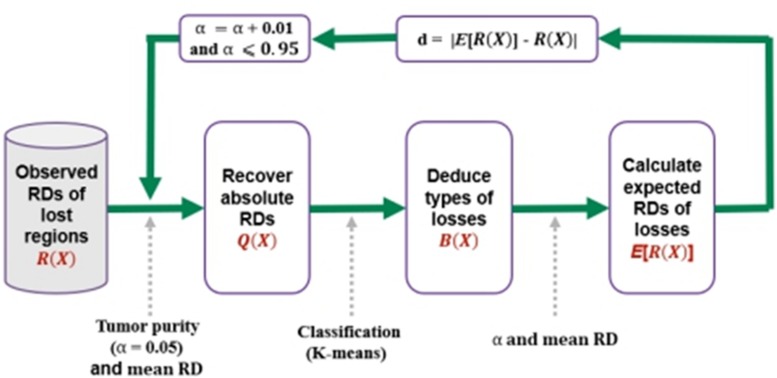
Procedure for the estimation of tumor purity. It starts with the input of observed RDs of lost regions, and recovers absolute RDs according to a given tumor purity and the mean RD across whole genome, and then performs a classification process to separate the losses into heterogeneous losses and homogeneous losses, and finally calculates the expected RDs of the losses. Here, *B*(*X*) is vector of 0/1, representing homogeneous and heterogeneous losses, respectively. The deviation between the expected and observed RDs is calculated, and the iterative process is continued by using a step of 0.01 for α until it is larger than 0.95.

For a clear understanding of the process, we depict the procedure in Figure 2. In the first step, it transfers the observed RDs of the copy number lost regions into absolute RDs according to the predefined tumor purity α, which is ranging from 0.05 to 0.95. In the second step, it clusters the lost regions into heterogeneous (one copy is lost) and homogeneous (two copies are lost) losses based on the absolute RDs, and denoting them by “1”s and “0”s, respectively. In the third step, it calculates expected RDs for the regions with heterogeneous or homogeneous losses, and calculates the deviation between the observed and expected RDs for the lost regions. Finally, the value of α that produces the smallest deviation is regarded as the estimated tumor purity. In the following subsections, the implementation of the above steps is described in detail.

#### Recovering Absolute RDs

The absolute RDs denote the RDs of pure tumor genome. Since such RDs are roughly linear to the amplitudes of CNVs, it is meaningful to make a recovery of absolute RDs before the classification of heterogeneous and homogeneous losses. Based on the observed RDs of the lost regions *R*(*X*) and the average RD across the whole genome to be analyzed $\bar{r}$, we recover the absolute RDs *Q*(*X*) using the Equation (1) given the tumor purity α. Here, it should be noted that the average RD $\bar{r}$ is assumed to be corresponding to the normal copy number. This is because CNVs usually accounts for a small fraction of the whole genome and include both directions (i.e., amplification and deletion) of copy number changes.

#### Deducing Types of Losses

Based on the recovered absolute RDs of lost regions, *Q*(*X*), we perform a classification process to separate them into two groups, i.e., heterogeneous and homogeneous losses. Here, we assume the ploidy of the tumor genome as 2. Thus, the heterogeneous losses will have one copy while the homogeneous losses will have zero copy. Currently, there are many algorithms that can perform such binary classification. For simplicity, we choose one of the most popular clustering algorithms, K-means algorithm, to carry out the classification by setting K to 2. One of the advantages of such algorithm is that it does not require a training process. Such advantage can facilitate the classification without the available of training data. Moreover, K-means is the most important flat clustering algorithm. Its objective is to minimize the average squared Euclidean distance of elements from their cluster centers, and the iterative process will be definitely converging (Selim and Ismail, 1984). The basic process of the algorithm is described as below.

1. Randomly selecting K centers from all the points in *Q*(*X*);
2. Traversing all the points in *Q*(*X*) and separating the points into the nearest central points;
3. Calculating the average value for each cluster and regarding each value as the new central point for the cluster;
4. Repeating steps 2-3 until the center values are at stable levels, or the predefined number of iterations have been performed.

**FIGURE 3:**
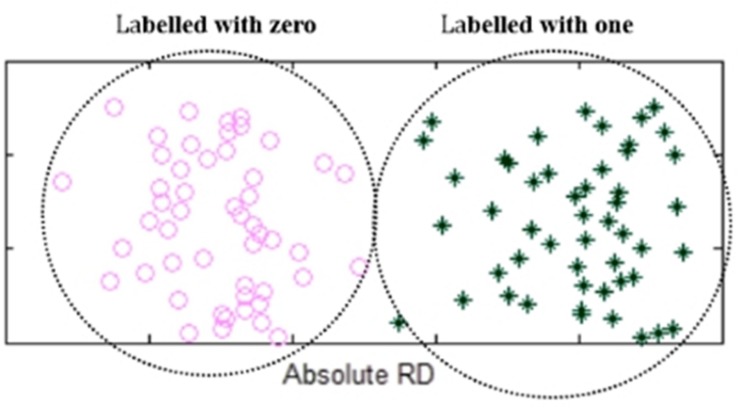
An example of showing the elements in the two groups of *Q*(*X*) that are labeled with zeros or ones. Absolute RDs of the lost regions are clustered into two groups by using K-means algorithm, where the left-side group is labeled with zeros while the right-side group is labeled with ones.

After the classification of the absolute RDs, the elements in the two groups of *Q*(*X*) are labeled as ones or zeros, representing heterogeneous losses or homogeneous losses. An example is shown in Figure 3. For convenience of the subsequent calculation, we use a 0/1 vector *B*(*X*) to denote all the elements in *Q*(*X*). Theoretically, the transformation from *Q*(*X*) to *B*(*X*) is a non-linear transformation, which is similar to the activation functions commonly used in neural network algorithms, such as sigmoid and ReLU functions. Moreover, the non-linear transformation is reasonable since the observed RDs are not strictly linear to the copy numbers in the lost regions.

#### Calculating Deviation Between the Observed and Expected RDs for the Lost Regions

Based on the non-linearly transformed vector *B*(*X*), we calculate the expected RDs for the lost regions. The calculation is expressed in Equation (3), where α is a given tumor purity value and $\bar{r}$ is the average RD across the whole genome.

(3) $$E\,\left[R\,\left(X\right)\right]=\frac{\alpha \,\bar{r}\,B\,\left(X\right)}{2}+\left(1-\alpha \right)\,\bar{r}$$

The deviation between the observed and expected RDs for the lost regions is then calculated by using Equation (4), where *n* denotes the total number of regions with either heterogeneous losses or homogeneous losses, |*E*[*R*(*X*)]−*R*(*X*)| denotes the absolute value of the average differences between the observed and expected RDs across all the elements in *X* (i.e., the set (*n*) of lost regions).

(4) $$d=\left|E\,\left[R\,\left(X\right)\right]-R\,\left(X\right)\right|=\frac{\sum_{x\in X}\left(\left|E\,\left[R\,\left(X\right)\right]-R\,\left(X\right)\right|\right)}{n}$$

**FIGURE 4:**
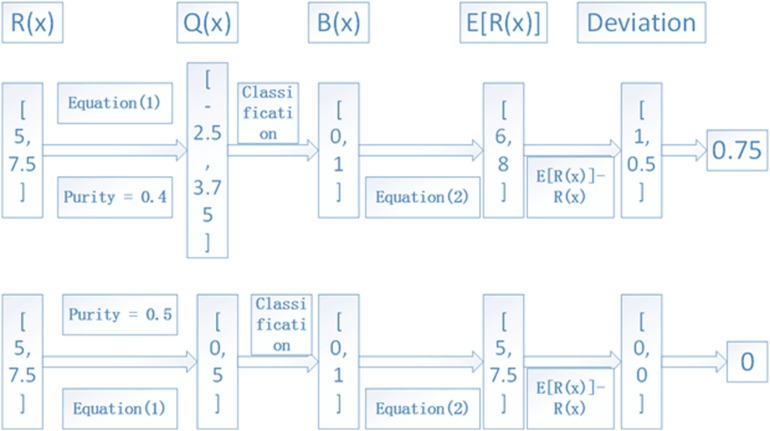
An example to show the calculation of the deviation between observed and expected RDs of lost regions. Here, two lost regions with RDs of 5 and 7.5 are assumed as the input, and α = 0.4 and 0.5 are used to show the deviation, and the average RD is 10.

The iterative process depicted in Figure 2 is continued with a step of 0.01 for the tumor purity α until it is larger than 0.95. The value that produces the minimum deviation will be regarded as the final tumor purity. For a clear understanding of the process, we depict an example in Figure 4, where we used two lost regions with observed RDs of 5 and 7.5 to show the calculation of deviations, and we find that α = 0.5 can produce the minimum deviation of zero.

### Inference of Absolute Copy Numbers

With the estimated tumor purity α, the absolute RDs of all the lost regions and gained regions could be inferred according to Equation (1). Here, the absolute RDs represent the RDs related with the pure tumor genome. Subsequently, the absolute copy numbers for each CNV region can be inferred by using Equation (5), where *x* denotes each element in the CNV regions including losses and gains, *Q*(*x*) denotes the corresponding absolute RD calculated by using Equation (1), and $\bar{r}$ denotes the average RD across the whole genome to be analyzed. This equation could be explained as: $\bar{r}$ is corresponding to the normal copy number, which is assumed to be 2, then the copy number of each CNV region can be deduced by using its absolute RD.

(5) $$C\,N=2\cdot \frac{Q\,\left(x\right)}{\bar{r}}$$

## Results

### Simulation Studies

Simulation study is a reasonable way to evaluate the performance of computational algorithms in CNV detection (Yuan et al., 2017). Here, we used one of the popular simulators, ART (Huang et al., 2012), to generate synthetic datasets by considering tumor purity ranging from 0.2 to 0.8. A set of CNVs assigned with different copy numbers (i.e., 0, 1, 4, 5, and 8) have been embedded into a background sequence. Here, we select the 21st chromosome from the reference HG19 as the background sequence. Sequencing reads with a uniform length of 100 base pairs have been generated based on such sequence, and the sequencing coverage depth is set to 10×. In each simulation configuration (i.e., each level of tumor purity), fifty replications of simulation datasets have been generated. This can help to improve the reliability of evaluation to the performance.

**FIGURE 5:**
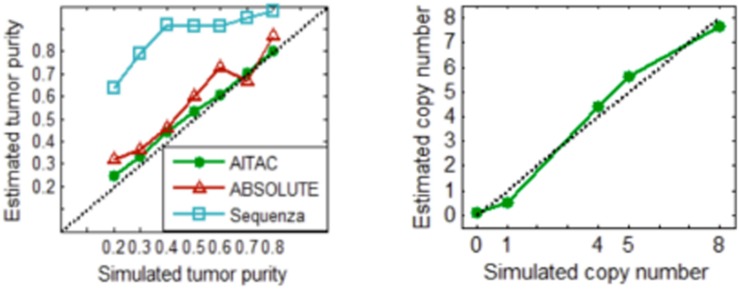
**(Left)** Comparison of the estimates of tumor purity between the three methods. **(Right)** Estimated copy numbers versus ground truth copy numbers. Here, the estimated values are the averaged values over the fifty replications in each simulation configuration.

With these simulation datasets, we performed the AITAC method and other two methods ABSOLUTE (Carter et al., 2012) and Sequenza (Favero et al., 2015) for a comparison. The comparative result is depicted in Figure 5, where the estimated tumor purity or copy number is an averaged value over the fifty replications in each simulation configuration. The result indicates that AITAC obtains the best estimation in terms of tumor purity, followed by ABSOLUTE, and then Sequenza. Since the two methods to be compared have not explicitly given the absolute copy numbers, we just compare our estimates with the ground truth copy numbers. Figure 5 (Right) shows that the estimates of AITAC are very close to the real ones. Such comparison demonstrates that our proposed method is valid and has the potential application ability in real datasets.

In addition, to show the performance of AITAC in estimating tumor purity based on the CNVs detected by other methods, we make a further comparison of tumor purity estimation to that by of detecting CNV by using the Control-FREEC method (Boeva et al., 2012). The comparative result is presented in the Supplementary Material, where we can see that the combination of AITAC and CNV_IFTV is superior to that of AITAC and Control-FREEC. This can be explained by that CNV_IFTV performs well in detecting CNVs and accurate detection of CNVs can help improving the estimation of tumor purity.

### Real Data Applications

**FIGURE 6:**
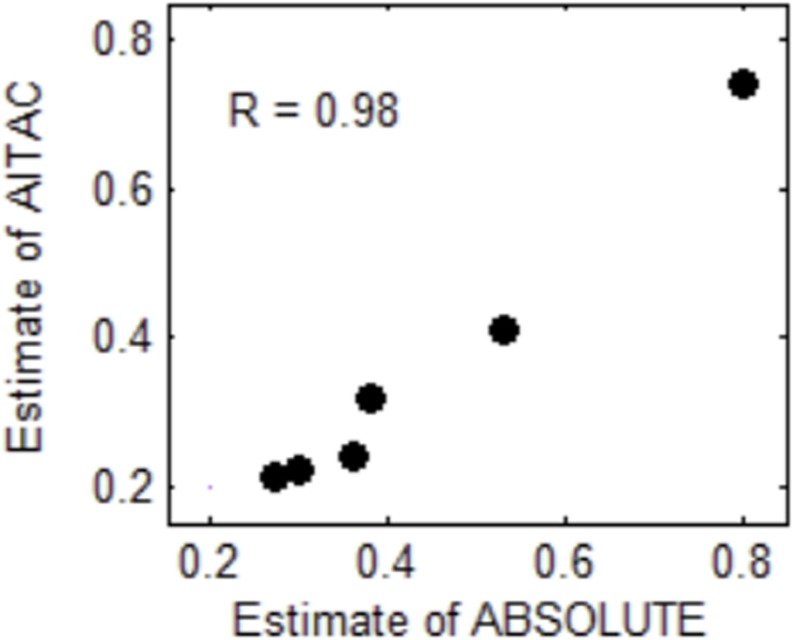
Comparison of tumor purity estimates between ABSOLUTE and AITAC. The correlation coefficient (R) between the two types of estimates is 0.98 for the six samples.

To examine the effectiveness of the AITAC method, we apply AITAC to a set of real sequencing samples obtained from the European Genome-phenome archive^1^ under accession No. of EGAD00001000144. These samples were sequenced from lung cancer patients and are formatted as BAM files. We performed the estimation of tumor purity on individual chromosomes and compared the result with that of ABSOLUTE. In Figure 6, we could note that the estimate of AITAC is very close to that of ABSOLUTE and the correlation coefficient (R) between them is 0.98. This implies that AITAC estimate is consistent with that of ABSOLUTE, which is the widely used tool for tumor purity estimation in copy number analysis. Thus, we may conclude that AITAC is a reliable method and will be expected as promising tool for the inference of tumor purity and absolute copy number in real tumor sequencing samples. In addition, we make a comparison of tumor purity estimation between AITAC, Sequenza, and ABSOLUTE with application to other samples. The corresponding result is presented in the Supplementary Material.

## Discussion

In this article, we proposed a new method, AITAC, for the inference of tumor purity and absolute copy numbers by using HTS data. It implements a pipeline analysis from an input of a sequencing sample and a reference genome to a report on CNVs, tumor purity and the corresponding absolute copy numbers. The most important feature of AITAC is that it models the relationship among observed RDs, expected RDs, and tumor purity in a non-linear way, and it does not rely on pre-detected mutation genotypes. We test the performance of AITAC on simulation and real sequencing datasets. The results demonstrate that AITAC is a valid and reliable method in the inference of tumor purity and absolute copy numbers.

There are still some disadvantages underlying the AITAC method. For example, it largely relies on the pre-detected CNV regions. If the CNV detection is not accurate, the tumor purity estimation will be influenced severely, and if the number of detected CNVs is small (e.g., there are only several deletions), the estimated result will be also influenced. For the future work, we plan to improve and extend the current version of AITAC from three perspectives. In the first place, we intend to incorporate tumor aneuploidy and heterogeneity in cancer samples to the copy number analysis pipeline. This will not only help to improve the accuracy of estimating tumor purity, but also help to provide more information for supporting cancer patient diagnosis. In the second place, extend the applications of AITAC to the analysis of single-cell sequencing data. This will help to discover novel cell-to-cell heterogeneity and the corresponding mutations (Vallejos et al., 2015; Zhang et al., 2016). In the last place, expect for the estimation of tumor purity from copy numbers, there many other types of information such as single nucleotide variations and methylation that can support for the tumor purity estimation. Thus, it will be a reasonable way to make an integrated analysis of multiple types of information for a more accurate estimation of tumor purity.

## Data Availability Statement

Publicly available datasets were analyzed in this study. This data can be found here: the European Genome-phenome archive (https://www.ebi.ac.uk/ega/home) under accession No. of EGAD00001000144.

## Author Contributions

XY and ZL participated in the design of algorithms and experiments. XY, ZL, and HZ combined the module of CNV detection into the whole framework of inferring tumor purity and absolute copy numbers. ZL implemented the python code. JB and JZ conceived of the study, participated in its coordination, and help edited the manuscript. All authors read the final manuscript and agreed the submission.

## Conflict of Interest

The authors declare that the research was conducted in the absence of any commercial or financial relationships that could be construed as a potential conflict of interest.

## Footnotes

1 https://www.ebi.ac.uk/ega/home
