# Supplementary material for: Accurate Inference of Tumor Purity and Absolute Copy Numbers From High-Throughput Sequencing Data
Source: Front Genet. 2020 Apr 30;11:458. doi: 10.3389/fgene.2020.00458 (PMC7205152; doi:10.3389/fgene.2020.00458)
Supplement: Supplementary file 1 [file Data_Sheet_1.docx]

1. Comparison of tumor purity estimation between the combination of AITAC and CNV_IFTV and the combination of AITAC and Control-FREEC.

The first step of AITAC in estimating tumor purity is the detection of copy number variations (CNVs). The more accurate detection of CNVs will be more helpful for the estimation of tumor purity. Since we have embedded the component of CNV detection module of CNV_IFTV to the AITAC method, it is necessary and meaningful to investigate the performance of estimating tumor purity by using other CNV detection methods. For this, we make a further comparison of tumor purity estimation to that by of detecting CNV by using the Control-FREEC method [[1](#_ENREF_1)]. The comparative result is presented in the Supplementary text, where we can see that the combination of AITAC and CNV_IFTV is superior to that of AITAC and Control-FREEC. This can be explained by that CNV_IFTV performs well in detecting CNVs and accurate detection of CNVs can help improving the estimation of tumor purity.


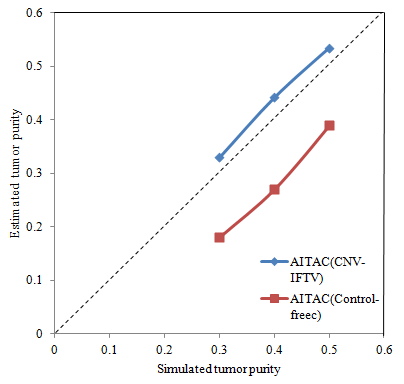


Supplementary Figure 1: Comparison of tumor purity estimation between the combination of AITAC and CNV_IFTV and the combination of AITAC and Control-FREEC

1. Tumor purity estimation of another sample (NA19238).


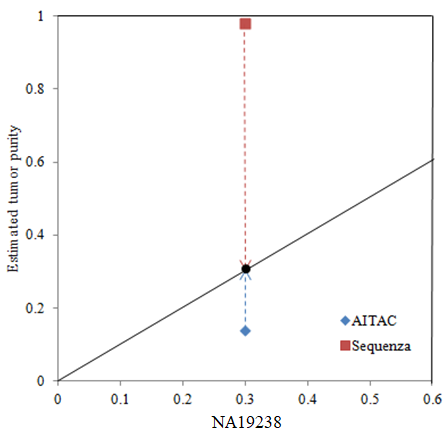


Supplementary Figure 2: Comparison of tumor purity estimates between AITAC and Sequenza with the reference of ABSOLUTE on the sample of NA19238. Here, the estimate of ABSOLUTE is 0.3.

[1] V. Boeva, T. Popova, K. Bleakley, P. Chiche, J. Cappo, G. Schleiermacher, I. Janoueix-Lerosey, O. Delattre, and E. Barillot, Control-FREEC: a tool for assessing copy number and allelic content using next-generation sequencing data. Bioinformatics 28 (2012) 423-5.
